# Cost implication of antibiotic-associated diarrhoea and the financial impact of probiotic use in its prevention

**DOI:** 10.1186/cc12021

**Published:** 2013-03-19

**Authors:** C Ebm, M Cecconi, C Moran, A Rhodes, T Rahman

**Affiliations:** 1 St Georges Healthcare Trust, London, UK

## Introduction

Antibiotic-associated diarrhoea (AAD) occurs in as many as 30% of patients receiving antibiotics, often leading to increased morbidity, prolonged in-hospital stay and additional healthcare resource utilisation. Age, antibiotics and prolonged postoperative ward and ICU stay have been suggested to be independent risk factors. In such patient populations, probiotics may be used to prevent antibiotic-associated diarrhoea, yet they are not routinely recommended as a component of perioperative care. The aim of this study was to model the long-term costs associated with AAD and to assess the effectiveness of probiotics as a preventive strategy.

## Methods

We developed a simulation model to determine clinical costs and outcomes attributable to AAD. To assess the cost-effectiveness of probiotics, as part of a perioperative regime, we constructed a decision tree. The model observes long-term costs and outcomes of probiotics as compared with conventional therapy, from a societal perspective. Input parameters, extracted from meta-analysis, clinical trials and national databases, include incidence numbers, costs and quality-adjusted health states for the remaining life (QALYs). Outcomes assessed were overall costs attributable to ADD and the cost-effectiveness of probiotics, described as costs/QALY.

## Results

Our results indicate an estimated incremental lifetime cost of £13,272.53 per ADD patient, largely driven by increased ICU length of stay and readmission rates. The addition of probiotics to the standard perioperative regime is associated with a small survival benefit of 1.2 months, yet a cost reduction of £917.3/ADD patient. The main cost was increased duration of ICU stay and readmissions, which contribute to 85% of total expenses.

## Conclusion

AAD is associated with a significant increase in costs from a societal perspective. The provision of probiotics can achieve substantial cost savings and can be recommended as a cost-effective regime in the perioperative setting. Preventing ADD offers a potentially significant reduction of in-hospital costs and resource expenditures.

